# The role and mechanism of claudins in cancer

**DOI:** 10.3389/fonc.2022.1051497

**Published:** 2022-12-22

**Authors:** De-Wen Wang, Wei-Han Zhang, Galiullin Danil, Kun Yang, Jian-Kun Hu

**Affiliations:** ^1^ Gastric Cancer Center and Laboratory of Gastric Cancer, Department of General Surgery, West China Hospital, Sichuan University, Chengdu, China; ^2^ State Key Laboratory of Biotherapy, West China Hospital, Sichuan University, Chengdu, China; ^3^ Central Research Laboratory, Bashkir State Medical University, Ufa, Russia

**Keywords:** claudin, signaling pathways, cancer, molecular targeted therapy, tumor diagnosis

## Abstract

Claudins are a tetraspan membrane protein multigene family that plays a structural and functional role in constructing tight junctions. Claudins perform crucial roles in maintaining cell polarity in epithelial and endothelial cell sheets and controlling paracellular permeability. In the last two decades, increasing evidence indicates that claudin proteins play a major role in controlling paracellular permeability and signaling inside cells. Several types of claudins are dysregulated in various cancers. Depending on where the tumor originated, claudin overexpression or underexpression has been shown to regulate cell proliferation, cell growth, metabolism, metastasis and cell stemness. Epithelial-to-mesenchymal transition is one of the most important functions of claudin proteins in disease progression. However, the exact molecular mechanisms and signaling pathways that explain why claudin proteins are so important to tumorigenesis and progression have not been determined. In addition, claudins are currently being investigated as possible diagnostic and treatment targets. Here, we discuss how claudin-related signaling pathways affect tumorigenesis, tumor progression, and treatment sensitivity.

## Introduction

Claudins (CLDNs) are a group of indispensable membrane tight junction (TJ) proteins that range in size from 17 to 27 kDa and include up to 27 members in mammals that exhibit a high degree of sequence homology (1). CLDNs were first identified in 1998 in a purified junctional fraction from chicken liver (2). According to hydrophilicity studies, claudin proteins comprise four transmembrane helices, two extracellular loops, an internal C-terminus, and a very short internal N-terminal region (2). The C-terminus contacts cytoplasmic proteins *via* a PDZ motif. Here, the first loop regulates paracellular charge selectivity, and the second loop of CLDN3 and CLDN4 is the receptor for a bacterial toxin (3–5). All known epithelial tissues exhibit tissue- and/or cell-specific expression of CLDNs. For instance, CLDN18.1 and CLDN18.2 are both particularly expressed in the stomach and lung alveolar epithelium, respectively (6, 7). CLDN3 is primarily expressed in alveolar epithelial type II cells (8). CLDN2, CLDN3, CLDN7, and CLDN15 are all found in high concentrations in intestinal epithelial cells (9, 10). CLDNs are generally located in the cell membrane and, under specific conditions, can be localized to the cytoplasm in both normal and neoplastic tissues. This cytoplasmic localization may be associated with CLDN phosphorylation and vesicle trafficking (11–14). The extracellular loops of CLDNs play an essential role in TJ formation by interacting with each other to seal the cellular sheet, thereby playing a role in regulating paracellular permeability and maintaining cell polarity (15). Growing evidence indicates that CLDNs are also involved in signal transduction by interacting with multiple proteins, regulating cell proliferation, cell growth, metabolism, metastasis and cell stemness. In addition, mutation of some CLDNs has been causally associated with human diseases, and CLDNs have been found to be deregulated in various cancers. This review aims to provide an overview of the potential signaling pathways and mechanisms of CLDNs (**Table 1**) in the occurrence, metastasis, and treatment of tumors.

**Table 1:** Role of claudins in cancers.

CLDNs type	Tumor type	CLDNs expression	Described mechanism	function	references
**CLDN1**	Breast cancer	↑	PI3K/Akt/mTOR signaling	upregulate CLDN1 expression	(16)
			ERK signaling	promote invasiveness and metastasis	(17)
	Colon adenocarcinoma	↑	EGFR/PKC/claudin-1 signaling	promote invasiveness and metastasis	(13)
			Wnt signaling	promote proliferation, invasiveness and metastasis; reduce anoikis	(18, 19)
			Smad4 inhibition	inhibit CLDN1 expression	(20)
			PI3K/Akt signaling	promote invasiveness and metastasis; reduce anoikis	(19)
	Stomach adenocarcinoma	↑	Wnt and PI3K/Akt signaling	promote proliferation, invasiveness and metastasis	(21)
	Hepatocellular carcinoma	↑	c-Abl-Ras-Raf-1-ERK1/2 signaling axis	promote cell stemness	(22)
	Pancreatic adenocarcinoma	↓	phosphorylates FAK and paxillin	promote invasiveness and metastasis	(23)
	Esophageal squamous cell carcinoma	↑	AMPK signaling	promote proliferation and metastasis	(14)
	Lung carcinoma	↑	PKCδ/iPLA2/PGE2/PPARγ signaling	regulate CLDN1 expression	(24)
**CLDN2**	Colorectal cancer	↑	MAPK signaling	regulate differentiation	(25)
			CLDN2/ZO1/ZONAB complex	promote proliferation and metastasis	(26)
			PI3K/Akt/NF-Kb signaling	upregulate CLDN2 expression	(27)
	clear cell renal cell carcinoma	↓	Hippo/Yap signaling	inhibit invasion and metastasis	(28)
	Osteosarcoma	↓	MAPK signaling	inhibit invasion and metastasis	(29)
	Breast cancer	↑	CLDN2/Afadin complex	promote metastasis	(30)
	Lung adenocarcinoma	↑	Decrease expression of resistance-associated protein/ABCC2	promote chemoresistance	(31)
**CLDN3**	Colorectal cancer	↑	ERK1/2 signaling	promote proliferation and metastasis	(32)
			PI3K/Akt signaling	promote proliferation and metastasis	(32)
			SCF/c-kit-JNK-AP-1 signaling axis	increase CLDN3 expression	(33)
		↓	Wnt signaling	induces de-differentiated; promote invasion and metastasis	(34)
	Lung adenocarcinoma	↑	EGF-activated MEK/ERK and PI3K-Akt pathways	promote proliferation, invasion, metastasis and chemoresistance	(35)
	Lung squamous cell carcinoma	↓	Wnt/β-catenin signaling	promote invasion and metastasis	(36)
	Hepatocellular carcinoma	↓	Wnt/βWnt/β-catenin-EMT axis	promote proliferation, invasion and metastasis	(37)
**CLDN4**	stomach adenocarcinoma	↓	PI3K/Akt signaling	promote proliferation and chemoresistance	(38)
	Renal cell carcinoma	Nuclear translocation	EphA2 and PKCϵ/CLDN4/hippo-Yap/EMT	promote invasion and metastasis	(39)
	Hepatocellular carcinoma	↑	Wnt signaling	promote proliferation	(40)
	Ovarian cancer	↑	Wnt signaling	promote proliferation, metastasis and chemoresistance	(41, 42)
	Uterine corpus endometrial carcinoma	↑	PI3K/Akt signaling	promote metastasis	(43)
**CLDN6**	Breast cancer	↓	p38 MAPK pathway	promote metastasis and chemoresistance	(42)
			beclin1-dependent autophagic cascade	inhibit invasion and metastasis	(44)
	stomach adenocarcinoma	↑	Hippo/Yap signaling	promote proliferation and invasion	(45)
	Hepatocellular carcinoma	↑	EGFR/AKT/mTOR signaling	promote proliferation, invasion and metastasis	(46)
**CLDN7**	Lung cancer	↓	MAPK/ERK pathway	promote invasion and metastasis	(47)
			Caspase pathway	promote chemoresistance	(48)
	Salivary adenoid cystic carcinoma	↓	Wnt signaling	promote proliferation, invasion and metastasis	(49)
	Colorectal cancer	↓	Wnt signaling	promote stemness	(50)
**CLDN10**	Osteosarcoma	↑	JAK1/Stat1 signaling	promote invasion and metastasis	(51)
**CLDN17**	Hepatocellular carcinoma	↑	Tyk2/Stat3 signaling	promote invasion and metastasis	(52)
**CLDN18.1**	Lung cancer	↓	Hippo signaling	inhibit progenitor cell proliferation and tumorigenesis	(53)
			IGF-1R/Akt signaling	inhibit proliferation, invasion and metastasis	(54)
**CLDN18.2**	stomach adenocarcinoma	↑↓	PKC/MAPK/AP-1 signaling axis	upregulate CLDN18.2 expression	(55)
	Pancreatic adenocarcinoma	↑	PKC signaling	upregulate CLDN18.2 expression	(56)

## CLDN1

**Figure 1:**
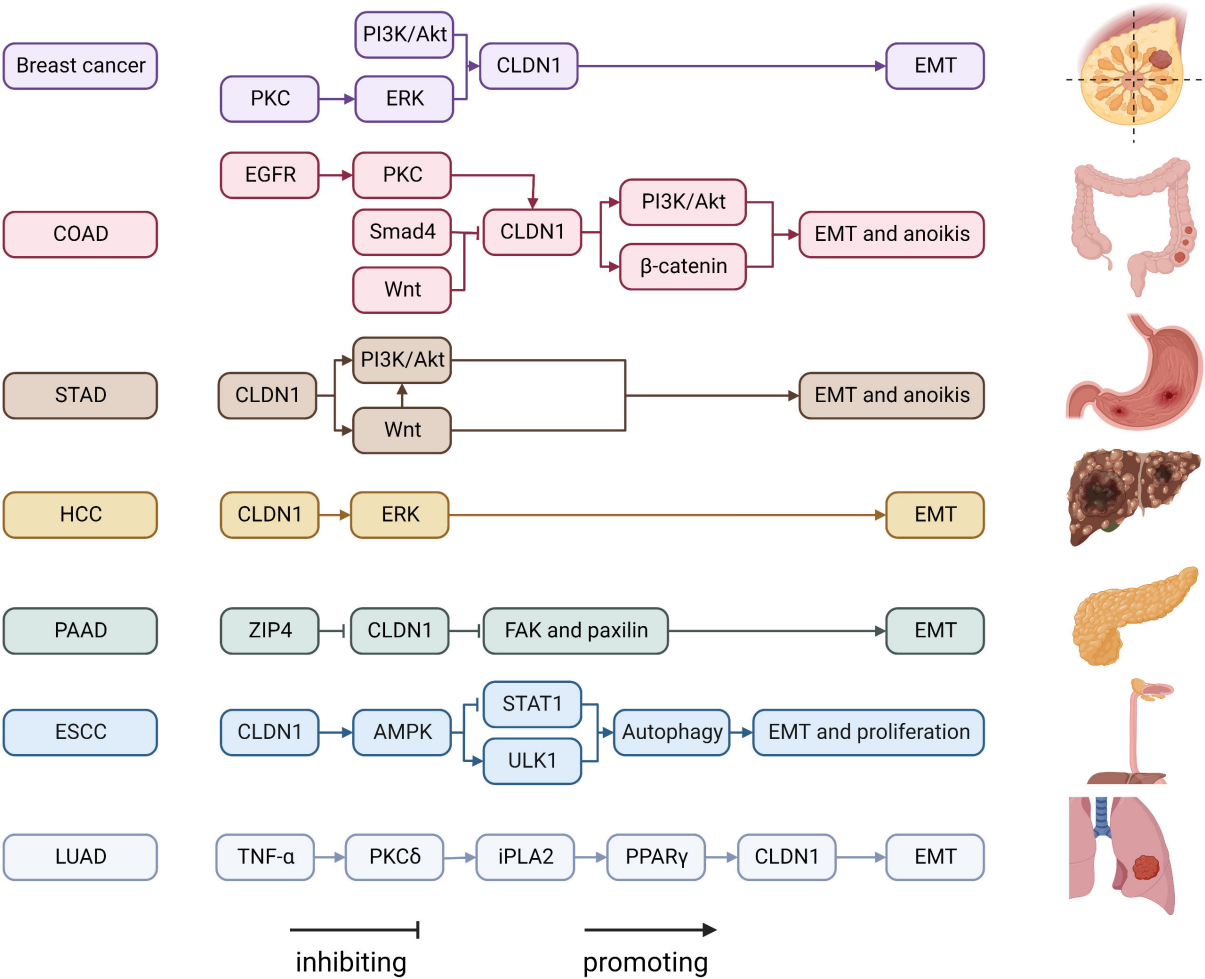
Regulatory mechanisms of CLDN1 expression in cancers. COAD, Colon adenocarcinoma; ESCC, esophageal squamous cell carcinoma; FAK, focal adhesion kinase; HCC, hepatocellular carcinoma; iPLA2, calcium-independent phospholipase A2; LUAD, lung adenocarcinoma; PAAD, pancreatic adenocarcinoma; PGE2, Prostaglandin E2; PI3K, phospatidylinositol-3 kinase; PKC, protein kinase C; PPAR-γ, Peroxisome proliferator-activated receptor-γ; STAD, stomach adenocarcinoma; STAT1, signal transducer and activator of transcription 1; TNF α, tumor necrosis factor α; ULK1, unc-51 like autophagy activating kinase 1; ZIP4, solute carrier family 39 member 4.

CLDN1 is one of the most-studied CLDNs in cancer (**Figure 1**). CLDN1 promotes the growth and progression of various tumors, including breast cancer, melanoma, oral squamous cell carcinoma, thyroid cancer, ovarian cancer, colon cancer, gastric cancer, hepatocellular carcinoma, and pancreatic cancer. CLDN1 is thought to be a tumor suppressor in lung and prostate cancers (57). In breast cancer, CLDN1 expression levels vary in pathology subtypes (58). In most breast cancers, downregulation of CLDN1 is more frequently correlated with higher invasiveness and poor prognosis (59). The basal-like subtype of breast cancer, which is typically associated with a poor prognosis, is one subtype where CLDN1 expression is elevated (60). At the molecular level, CLDN1 is regulated by disintegrin and metalloproteinase-15 (ADAM15) *via* PI3K/Akt/mTOR signaling in breast cancer (16). In addition, CLDN1 expression is also be upregulated through the ERK signaling pathway in the MCF7 cell line (17).

The role of CLDN1 expression in colon cancer is debated. While some studies link increasing CLDN1 expression to the growth and spread of colon cancer, others believe that low CLDN1 levels are a standalone predictor of a poor prognosis (61, 62). This disparity could be caused by Resnick’s (62) work mistakenly identifying nuclear translocation of CLDN1 as low expression of CLDN1, when in fact CLDN1 expression was high in metastatic colon cancer. The migration ability of colon cancer cells is enhanced by the upregulation and internalization of CLDN1 *via* the EGFR/PKC/CLDN1 signaling pathway (13). Additionally, CLDN1 can upregulate ZEB-1, which reduces E-cadherin expression in colon cancer cells, increases their invasive activity and decreases anoikis (19). Previous studies demonstrated that the Wnt signaling pathway promoted CLDN1 expression and enhanced colon cancer cell proliferation, invasion and metastasis (63–67). By increasing CLDN1 gene expression, the Wnt signaling pathway inhibitor FH535 prevented colorectal cancer cells from proliferating and migrating (18). In stomach adenocarcinoma, CLDN1 is an oncogenic molecule. CLDN1’s malignant potential may be attributed in part to its regulation of the Wnt signaling pathway (21) with its knockdown significantly inhibiting cell proliferation, migration, and invasion while increasing apoptosis (68). CLDN1 is highly expressed in hepatocellular carcinoma and acts as a promoter of epithelial-to-mesenchymal transition (EMT) through the c-Abl-Ras-Raf-1-ERK1/2 signaling axis (22, 69, 70). It has been demonstrated that CLDN1 downregulation in pancreatic cancer results in the phosphorylation of FAK and paxillin, which further promotes cell invasion, migration, and tumor metastasis (23). Given that CLDN1 is mostly found in the nucleus of esophageal squamous cell carcinoma (ESCC) and has been shown to be abnormally elevated, CLDN1 promotes ESCC growth and metastasis by upregulating the expression of ULK1 *via* the AMPK/STAT1 signaling pathway (14). Low CLDN1 expression in response to TNF-α is regulated by the PKC/iPLA2/PGE2/PPAR signaling cascade and is associated with lung adenocarcinoma rather than lung squamous cell carcinoma, whereas increased CLDN1 expression is associated with a better prognosis and tumor suppressive activity (24, 71, 72). CLDN1 is involved in a variety of signaling pathways that are particularly involved in invasion and migration. Numerous CLDN1-targeting antibodies, including 3A2 and 6F6, have been demonstrated to be tumor suppressive (73, 74). As a result, the molecules in the pathway that interact with CLDN1 can be investigated further as potential drug targets.

## CLDN2

According to a growing body of evidence, dysregulated CLDN2 expression affects processes underlying carcinogenesis and metastasis formation, such as proliferation, migration, and EMT (29, 75–80). Increased CLDN2 expression is mediated by the EGF/Ras/Raf/MEK/ERK/c-Fos pathway and the PI3K/Akt/NF-B pathway (25, 27, 75, 81–85). Loss of CLDN2 promotes the dissociation of the CLDN2/ZO1/ZONAB complex, which then induces ZONAB translocation to the nucleus and enrichment at the NDRG1 promotor to activate its transcription, thereby preventing the growth and spread of colorectal cancer (26, 86–91). Through its PDZ binding motif, CLDN2 interacts with YAP in renal clear cell carcinoma and prevents YAP nuclear localization and activation (28). Osteosarcoma tissue exhibits reduced levels of CLDN2 expression, thereby inhibiting the Ras/Raf/MEK/ERK signaling pathway *via* afadin and eventually reducing the capacity of osteosarcoma cells to migrate (29). However, the effective development of breast cancer metastases is made possible by signaling downstream from a CLDN2/Afadin complex (30). Notably, increased intracellular accumulation of anticancer agents and decreased expression of ABCC2 were observed in CLDN2-downregulated lung adenocarcinoma cells (31). Hence, inhibiting CLDN2-related signaling pathways could represent a potential novel target for therapeutic intervention. In fact, flavonoids, such as quercetin, chrysin, kaempferol, and luteolin as well as inhibitors of intracellular signaling reduce chemoresistance, invasion, and proliferation in a variety of malignancies (82, 92). VPDSM and DSMKF, short peptides that mimic CLDN2’s second extracellular loop (ECL2), may directly act on the CLDN2 protein and lead to CLDN2 internalization, whereas CLDN2 expression is reduced *via* the clathrin-dependent endocytosis pathway (93, 94). In xenografted mice, CLDN2 ECL-targeting antibody 1A2 suppresses tumor growth in a manner similar to CLDN1-targeting antibody 3A2 (95). Inhibitors targeting CLDN2-related intracellular signal transduction molecules inhibit cancer development. The idea of reducing CLDN2 expression in cells by mimicking the short peptide of CLDN2’s second extracellular loop (ECL2) to improve tumor cell sensitivity to chemotherapy drugs provides us with new inspiration. We consider whether this drug design concept can be applied to other CLDNs.

## CLDN3

Numerous human tumors and their subsequent metastasis have been linked to the dysregulation of CLDN3. CLDN3 regulation involves a variety of signaling pathways. Studies have revealed that SCF/c-kit signaling exclusively enhances CLDN3 expression in colorectal cancer, mainly by activating the JNK pathway, and that the SCF/c-kit-JNK-AP-1 signaling axis is crucial for controlling CLDN3 expression (33). EGF-induced CLDN3 upregulation *via* the ERK pathway and PI3K-Akt pathway is associated with increased malignancy in HT-29 colon cancer cells (32). Another contentious finding, however, demonstrated that CLDN3 is the most abundant cell-adhesion protein in the normal colon and that its absence promotes Wnt signaling, which is subsequently excessively triggered by IL-6/gp130/STAT3 signaling (34). Contradictory findings from these two studies on the involvement of CLDN3 in colorectal cancer indicate that we still know very little about the role of CLDN3 in colorectal cancer. In addition to what is already known, CLDN3 overexpression or silencing alter the structure and function of TJs, hence disrupting intestinal homeostasis. CLDN3 expression is increased in lung adenocarcinoma compared with lung squamous cell carcinoma (96). Through Wnt/β-catenin signaling pathway inhibition, CLDN3 prevents EMT in lung squamous cell carcinoma (36). However, CLDN3 overexpression in lung cancer can increase malignant potential by activating the EGF-activated MEK/ERK and PI3K-Akt pathways (35). The reasons for this discrepancy are unclear but may be attributed to the different properties of tumorigenicity. Type II alveolar epithelial cells are the primary lung cells that express CLDN3 (97). Lung adenocarcinoma is thought to develop from type II alveolar epithelial cells, whereas lung adenosquamous carcinoma and lung squamous cell carcinoma develop from different epithelial cell components (98). For primary hepatocellular carcinoma patients, downregulation of the CLDN3-Wnt/β-catenin-EMT axis is significantly correlated with survival differences (37). CLDN3 is associated with drug resistance and prognosis in a number of tumors, and its role involves multiple signaling pathways, including the PI3K signaling pathway, ERK signaling pathway, and Wnt signaling pathway. CLDN3 and CLDN4 have been identified as useful biomarkers in the diagnosis of gynecological malignancies and can be combined with other markers such as CA125. CLDN3 and CLDN4 have been demonstrated to be effective Clostridium perfringens enterotoxin (CPE) targets in the treatment of gynecological malignancies (99).CPE’s C-terminal domain(c-CPE) can bind to the second extracellular loop of a variety of claudin proteins to form hexameric pores at the membrane surface, resulting in calcium influx and cell death (100).CLDN3, 4, 5, 6, 7, 8, 9 and 14 are examples of claudin proteins that have been found to bind to CPE. As a result, Claudius exhibit considerable potential as a cancer therapy targets.

## CLDN4

CLDN4 expression is dysregulated in multiple cancers, including esophageal, gastric, breast, lung, biliary, ovarian, endometrial, bladder, uterine, renal, nasopharyngeal, and prostate cancers (101–112). EGF enhances the degradation of CLDN4 at the posttranslational level *via* MEK/ERK signaling and PI3K/Akt signaling (113). Furthermore, PI3K/Akt signaling is activated by reduced CLDN4 expression (38, 114). TGF-β and SMAD2/3/4 signaling regulate CLDN4 promoter activity, and expression levels differ between cancers (115, 116). CLDN4, which does not form TJs, participates in intracellular signaling (117). EphA2 and PKC phosphorylation of CLDN4 may weaken TJs, release CLDN4 from TJs, and boost CLDN4’s ability to bind to YAP and ZO-1 to create a nuclear translocating complex, which would activate YAP and EMT (39, 118). CLDN4 upregulation also activates Wnt/β-catenin signaling and triggers the progression of hepatocellular carcinoma and ovarian cancer (40–42). In uterine corpus endometrial carcinoma, CLDN4 induces tumorigenesis by activating the PI3K/AKT pathway (43). CLDN4 is commonly overexpressed in many epithelial cancers. As previously stated, CLDN4 has sparked considerable interest due to its role as a natural CPE receptor. A C-terminal fragment containing only CPE (c-CPE) can bind specifically to claudin protein and increase paracellular permeability by disrupting TJs, improving drug delivery across tissue membranes without disrupting plasma membrane integrity and causing cytotoxicity (119). This finding indicates that CPE represents an effective drug carrier and is less toxic than natural CPE, making it more suitable for cancer treatment. Currently, some drugs designed with c-CPE characteristics have been used in clinical research with good results, such as Doxorubicin-loaded C-SNPs (DOX-C-SNPs) and poly(lactic-co-glycolic-acid) (PLGA) nanoparticles (NPs) modified with the carboxy-terminal binding domain of CPE (c-CPE-NPs) (120, 121) c-CPE 194 is a c-CPE mutant that only binds to Claudin-4 and improves the efficacy of anticancer agents (122). Furthermore, many studies have shown that combining c-CPE and chemotherapy drugs can increase tumor cell sensitivity to chemotherapy drugs (123). A monoclonal antibody that recognized the ECL1 of CLDN3 and CLDN4, KM3907 (IgG2a), induced antibody-dependent cellular cytotoxicity and complement-dependent cytotoxicity *in vitro* and significantly inhibited tumor formation in SCID mice (124). Finally, CLDN4 exhibits significant therapeutic and diagnostic utility.

## CLDN6

**Figure 2:**
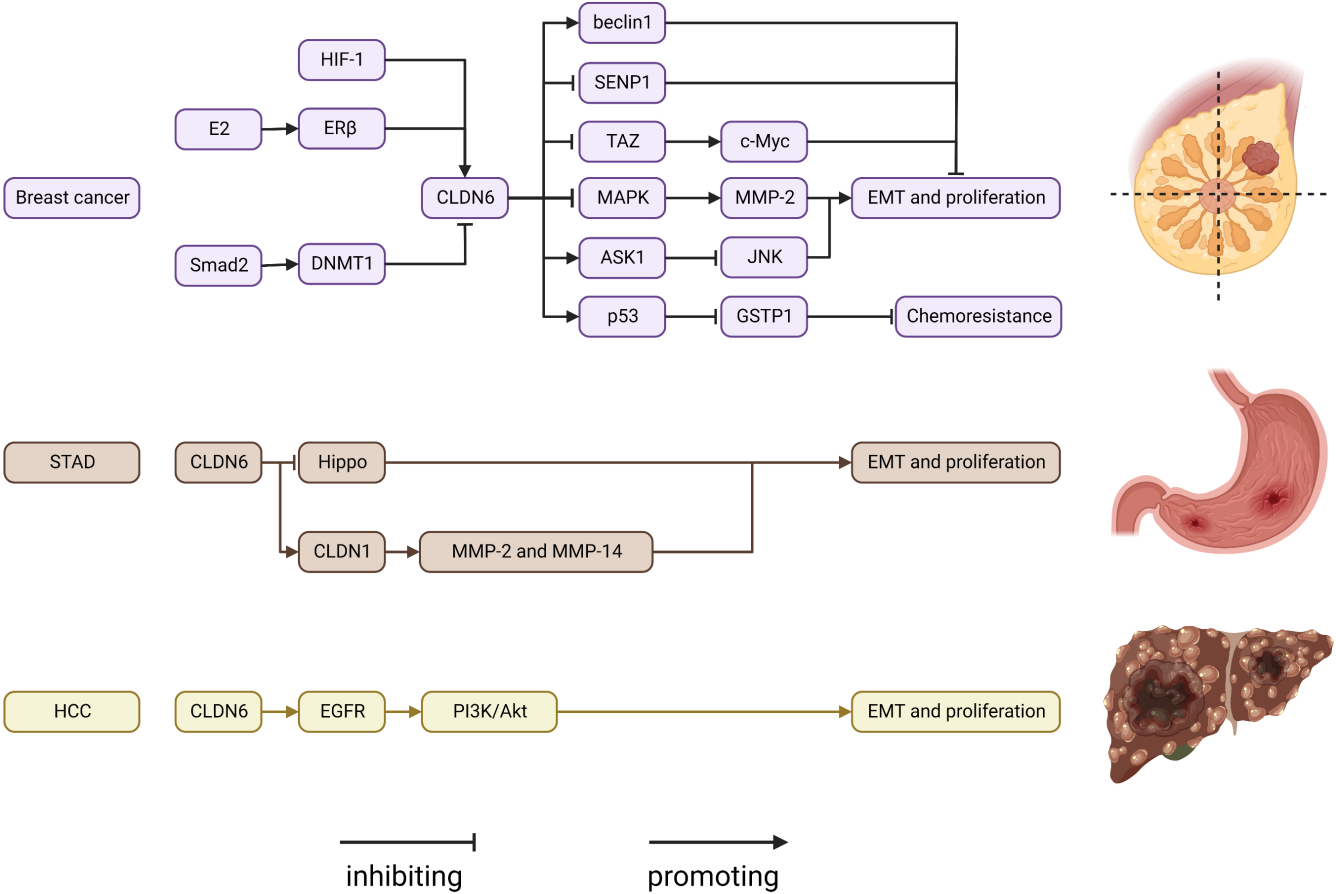
Regulatory mechanisms of CLDN6 expression in cancers. ASK1, apoptosis signal-regulating kinase 1; CLDN, claudin; DNMT1, DNA methyltransferase 1; E2, 17β-estradiol; EGFR, epidermal growth factor receptor; ERβ, estrogen receptor β; GSTP1, glutathione S-transferase-p1; HIF-1, hypoxia-inducible factor 1; MMP, matrix metalloproteinase; SENP1, SUMO-specific protease 1; STAD, stomach adenocarcinoma; TAZ, transcriptional coactivator with PDZ-binding motif.

CLDN6 is described as a tumor suppressor gene and helps maintain cell stemness. CLDN6 expression levels are altered in various types of cancer (**Figure 2**). CLDN6 is well studied in breast cancer. Compared with adjacent normal tissues, CLDN6 expression levels are significantly reduced in breast cancer tissues (125, 126). It has been proposed that CLDN6 silencing is associated with DNA methyltransferase 1 (DNMT1)-induced DNA methylation, which is controlled by the TGF-β/SMAD2 pathway (127). By bringing MeCP2 to the CLDN6 promoter, deacetylating H3 and H4, and changing the chromatin structure, DNA methylation inhibits the production of CLDN6 (128). Silencing the CLDN6 gene causes increased growth and migratory ability, as well as increased MMP-2 expression and activity, which may be mediated by the MAPK pathway (129). CLDN6 expression is linked to apoptosis signal-regulating kinase 1 (ASK1) expression, and restoring CLDN6 expression in breast cancer cells reduces ASK1 phosphorylation, activates the downstream target proteins JNK and p38 kinase, and induces apoptosis (130–133). 17β-Estradiol (E2) enhances CLDN6 expression *via* estrogen receptor α (ERα) and estrogen receptor β (ERβ) (44, 134). In addition, CLDN6, which triggers the beclin1-dependent autophagic cascade, is a mediator of the inhibitory effect of ERβ on breast cancer cells migration and inveations (44). The results from studies on the sensitivity of conventional therapeutic drugs have demonstrated that the reinstatement of CLDN6 expression in breast cancer cells increases chemoresistance to Adriamycin, an anticancer medication that is frequently used to treat breast cancer by activating the AF-6/ERK signaling pathway and upregulating cancer stem cell properties (135, 136). CLDN6-induced chemoresistance in breast cancer is mediated by glutathione S-transferase-p1 (GSTP1), which is regulated by p53 (137). CLDN6 binds to the transcriptional coactivator with a PDZ-binding motif (TAZ) and reduces the amount of TAZ. This activity inhibits the transcription of c-MYC, which slows the growth of breast cancer cells (138). Under hypoxic conditions, the accumulation of hypoxia-inducible factor 1 (HIF-1) promotes CLDN6 transcription, and increased CLDN6 weakens HIF-1α protein stability by reducing SUMO-specific protease 1 expression and preventing the deSUMOylation of HIF-1α, ultimately leading to HIF-1α degradation and breast cancer metastasis suppression (139).

Compared to histologically normal gastric tissues, CLDN6 expression is noticeably reduced in the majority of gastric cancer tissues; however, some have remarkably greater expression levels (140–143). Additionally, CLDN6 interacts with LATS1/2 to reduce LATS1/2 and YAP1 phosphorylation, influence YAP1 entry into the nucleus, and enhance the invasive ability of gastric cancer cells (45, 144). Through the expression of CLDN1 on the membrane, CLDN6 can also cause MMP-2 activation, which encourages cell migration and invasion (145). However, another study found the converse to be true; specifically, CLDN6 overexpression caused differentiation in BGC-823 cells by blocking the JNK pathway with no impact on *in vitro* apoptosis, proliferation, invasiveness, or migration (143). The different gastric cancer cell lines that were used might be the cause of the inconsistent results. CLDN6 is highly expressed in hepatocellular carcinoma, and can promote EMT in hepatoma cells (146). In addition, CLDN6 overexpression may act as an oncogene and enhance HepG2 cell migration, invasion, and proliferation *via* EGFR/AKT/mTOR signaling in hepatocellular carcinoma (46). Chimeric antigen receptor (CAR)-T/NK and antibody−drug conjugates are two cutting-edge and effective therapy options for cancer. CLDN6 was used as a promising target in the development of treatments. AMG 794 for nonsquamous non-small cell lung cancer or epithelial ovarian cancer (NCT05317078) as well as DS-9606a for advanced solid tumors are currently being assessed in two phase 1 studies (NCT05394675) (147, 148). Additionally, research on CLDN6-CAR-NK-cell treatment for advanced solid tumors (NCT05410717) is ongoing (149). The results from the aforementioned CLDN6 studies are anticipated.

## CLDN7

CLDN7 is either downregulated or disrupted in a variety of tumors. CLDN7 downregulation may lead to epithelial damage and TJ structural changes, which are associated with the etiology and progression of malignancies (150–154). However, CLDN7 was strongly expressed in benign bronchial epithelial cells but substantially expressed or completely absent in lung cancer cells. This finding suggests that CLDN7 may act as a tumor suppressor in lung cancers. CLDN7 transfection induces the downregulation of ERK1/2 phosphorylation, leading to MAPK/ERK pathway inhibition. MAPK/ERK pathway inhibition causes the formation of shorter foot processes, thereby decreasing cellular motility (47). CLDN7 is phosphorylated by protein kinase C at serine 204, and CLDN7 phosphorylation increases chemosensitivity to cisplatin treatment by activating the caspase pathway in lung cancer cells (48). CLDN7 expression in salivary adenoid cystic carcinoma was lower than that in paired normal tissues, and CLDN7 knockdown promotes cell proliferation and metastasis through the Wnt/β-catenin signaling pathway in SACC-LM cells (49). CLDN7 expression is downregulated as colorectal cancer tissue differentiation grade decreases, the invasion and migration abilities of colorectal cancer cells are subsequently enhanced as a result of modulation of CLDN7 expression (155, 156). The stemness properties of colorectal cancer are enhanced by CLDN7, which mediates Wnt/β-catenin pathway activation by SOX-9 (50, 157, 158). CLDN7 expression is involved in a variety of activities and is a crucial target for preventing tumor proliferation and invasion. Early detection, treatment, and prognosis of cancer may be improved through research focusing on CLDN7.

## CLDN10

Multiple groups have reported that the CLDN10 expression level is associated with the progression of a variety of tumors and may serve as a potential prognostic biomarker (159–164). CLDN10 is highly expressed in osteosarcoma cells compared with fetal osteoblast cells, and CLDN10 overexpression in osteosarcoma cells enhances the JAK1/STAT1 signaling pathway to significantly promote proliferation and motility (51). However, the underlying mechanisms have received little attention, and more research is needed.

## CLDN17

Tumor-related mechanisms in signaling events related to CLDN17 have been less studied. CLDN17 overexpression is strongly linked to cancer metastasis and survival rate in patients with hepatocellular carcinoma and promotes cell migration and invasion in the hepatocyte line HL7702. Tyk2/STAT3 signaling may be one of the most important mechanisms (52). In contrast, CLDN17 may serve as a negative regulator in oral cancer by blocking invasion and migration *via* the EMT process (165). More research is needed to determine how CLDN17 contributes to the pathogenesis of cancer.

## CLDN18

**Figure 3:**
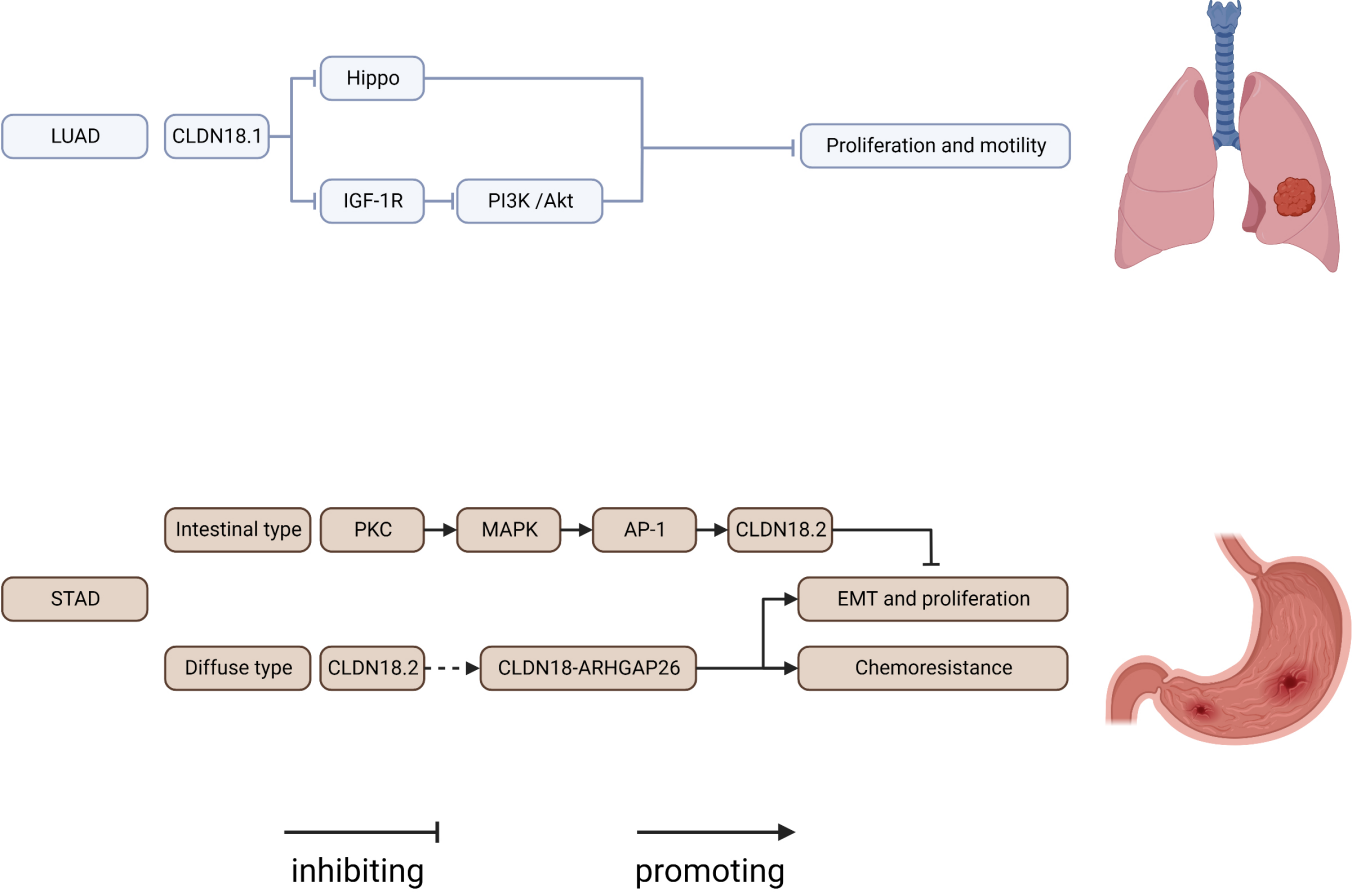
Regulatory mechanisms of CLDN18 expression in cancers. AP-1, AP1 transcription factor; IGF-1R, insulin-like growth factor 1 receptor; LUAD, lung adenocarcinoma; PI3K, phospatidylinositol-3 kinase; PKC, protein kinase C; STAD, stomach adenocarcinoma.

CLDN18 has received the most attention in recent years (**Figure 3**). It has two tissue-specific isoforms, each with its own promoter: exon 1 and common exons 2–5. CLDN18.1 is exclusively expressed in lung epithelium, whereas CLDN18.2 is only found in the stomach and bone. CLDN18.1 is aberrantly localized in many cancers, especially pancreatic and gastric cancers (7, 55, 166–170).

CLDN18.1 knockout resulted in increases in lung size and cellularity due to alveolar epithelial type II cell proliferation in mice. Furthermore, most CLDN18 knockout mice develop lung adenocarcinomas spontaneously between the ages of 18 and 20 months (53, 171–173). CLDN18.1 inhibits two oncogenic pathways, Yap/Taz and IGF-1R signaling, resulting in the suppression of cell motility and increased cell death in anoikis by inhibiting PDK1 and subsequent inhibition of Akt phosphorylation (53, 54, 174).

CLDN18.2 represents a promising new therapeutic target for pancreatic and stomach cancer. Downregulation of CLDN18.2 is linked to the intestinal phenotype of gastric cancers, and CLDN18.2 expression in gastric cells is upregulated *via* a PKC/MAPK/AP-1-dependent pathway (55). CLDN18.2 is highly expressed in gastric signet-ring cell carcinoma (175). According to reports, genomically stable gastric cancers frequently have chromosomal rearrangements of the *CLDN18* and *ARHGAP* genes, primarily *CLDN18-ARHGAP26/6* fusions. Importantly, after *CLDN18-ARHGAP26* was introduced, considerable resistance to 5-fluorouracil and oxaliplatin was observed in a number of gastric cancer cell lines, which may account for the poor drug response of patients harboring this fusion product (176–180). The relationship between CLDN18.2 expression and the prognosis of gastric cancers remains controversial. Past research indicated that CLDN18.2 was a good marker of poor survival in gastric cancer and that the downregulation of CLDN18 may represent an early event of gastric carcinogenesis with an intestinal phenotype (181, 182). However, resent research revealed that CLDN18.2 expression was not related to prognosis among patients with gastric cancer (183–186). The diverse ethnic makeup of the cohort, intra-tumor variation and the limitations of a limited sample size can all be used to explain this variation in CLDN18 expression rate. The use of different CLDN18 expression detection antibodies can also result in variations in CLDN18 expression rates. For the creation of comparable data in future research, uniform immunostaining and grading methodologies are therefore crucial. CLDN18.2 is ectopically highly expressed in pancreatic cancer, and activation of the PKC pathway might be involved (56, 187). CLDN18 has been considered a potential marker and therapeutic target in gastric-type mucinous carcinoma, esophageal adenocarcinoma, mucinous ovarian cancer, ductal pancreatic adenocarcinoma, and intrahepatic cholangiocarcinoma (170, 188–190). The creation of new medications and cutting-edge therapies for CLDN18.2 has recently become a popular area of study. Zolbetuximab (IMAB362), a monoclonal antibody targeting CLDN18.2, extends progression-free and overall survival in CLDN182-prositive patients with gastric and gastroesophageal adenocarcinoma (191–193). In addition, patients with advanced gastric cancer also benefited from CLDN18.2-targeted CAR-T cells (CT041) in a phase 1 trial (194). We are eagerly waiting for the results of a number of ongoing prospective clinical studies assessing CLDN18.2 in solid tumors. In conclusion, CLDN18 is a very good target for determining what is wrong with a tumor and treating it.

## Conclusion

Since the discovery of the claudin protein, a great deal has been learned regarding its structure and function. On the one hand, the claudin family of membrane proteins interacts with the zonula occludens (ZO) family, junctional adhesion molecules (JAMs), and membrane lipids to play a central role in the structure and function of tight junctions. In MDCK II cells, Claudin-1/2/3/4/7 quintuple-KO (claudin quintuple-KO) cells lack TJ chains, but the adjacent plasma membranes remain tightly attached to each other, showing only disruption of the permeability barrier to ions and small molecules. However, when JAM-A was further removed from claudin quintuple-KO MDCK II cells, the intercellular space was enlarged, and the macromolecular permeability barrier was disrupted (195).These phenomena indicate that claudin proteins play a decisive role in the formation of TJs and affect the function of the permeability barrier together with cell membrane molecules, such as JAMs. When the expression of claudin protein is altered or translocated, the structure or function of TJs is damaged, and barrier permeability changes, resulting in homeostasis dysregulation. TJ results and functional changes also weaken the cell anchor, making tumor cells more prone to invasion and migration. On the other hand, Claudin proteins are an important component of cell signaling that is involved in several classic tumor-related signaling pathways and affects every aspect of tumor biology and all stages of tumor development. In addition, the tissue-specific expression of CLDNs and the disruption of the tight junction structure in malignant tissues suggest that CLDNs may represent ideal diagnostic and therapeutic targets (e.g., CLDN18.2 for gastric cancer, c-CPE for breast cancer and pancreatic cancer). As more research is conducted, we will learn more about their role in tumorigenesis, which will lead to the development of novel cancer treatments and prevention strategies.

## Author contributions

All authors listed have made a substantial, direct, and intellectual contribution to the work and approved it for publication.

## Glossary

**Table:**

ABCC2	multidrug resistance protein 2
AF-6	afadin 6
AP-1	AP1 transcription factor
ASK1	apoptosis signal-regulating kinase 1
COAD	Colon adenocarcinoma
DNMT1	DNA methyltransferase 1
DNMT1	DNA methyltransferase 1
E2	17β-estradiol
ECL	extracellular loop
EGFR	epidermal growth factor receptor
EMT	Epithelial-to-Mesenchymal Transition
EphA2	ephrinA2 receptor
ERα	estrogen receptor α
ERβ	estrogen receptor β
FAK	focal adhesion kinase
gp130	glycoprotein-130
GSTP1	glutathione S-transferase-p1
HCC	hepatocellular carcinoma
HIF-1	hypoxia hypoxia-inducible factor 1
IGF-1R	insulin-like growth factor 1 receptor
IL-6	interleukin-6
iPLA2	calcium-independent phospholipase A2
JNK	c-Jun N-terminal kinase
LATS1/2	large tumor suppressor 1 and 2
LUAD	lung adenocarcinoma
MAPK	Mitogen-activated protein kinase
MeCP2	methyl-CpG-binding protein 2
MEK	Mitogen-activated protein kinase
MMP-2	matrix metalloproteinase-2
mTOR	mechanistic target of rapamycin
NDRG1	N-myc downstream regulated gene-1
NF-κB	nuclear factor-κB
PAAD	pancreatic adenocarcinoma
PDK1	3- phosphoinositide-dependent protein kinase 1
PGE2	Prostaglandin E2
PI3K	phospatidylinositol-3 kinase
PKC	protein kinase C
PKM2	pyruvate kinase M2
PPARγ	Peroxisome proliferator-activated receptor-γ
SCF	stem cell factor
SENP1	SUMO-specific protease 1
STAD	stomach adenocarcinoma
STAT3	signal transducer and activator of transcription 3
SUMO	small ubiquitin-related modifiers
TAZ	transcriptional coactivator with PDZ-binding motif
TGF-β	Transforming growth factor-β
TJ	tight junction
TNF α	tumor necrosis factor α
Tyk2	tyrosine kinase 2
ULK1	unc-51 like autophagy activating kinase 1
YAP	Yes-associated protein
ZEB1	zinc-finger E homeobox-binding protein 1
ZIP4	solute carrier family 39 member 4 ZO1, zona occludens 1
ZONAB	(ZO-1)–associated nucleic acid binding protein
